# Circulating proteins associated with histological subtypes of lung cancer from genetic and population-based perspectives

**DOI:** 10.1371/journal.pgen.1011821

**Published:** 2025-08-25

**Authors:** Zhangyan Lyu, Guojin Si, Mengbo Xing, Wenxuan Li, Ximin Gao, Meng Wang, Fengju Song, Kexin Chen

**Affiliations:** 1 Department of Epidemiology and Biostatistics, Key Laboratory of Molecular Cancer Epidemiology, Key Laboratory of Prevention and Control of Human Major Diseases, Ministry of Education, Tianjin’s Clinical Research Center for Cancer, National Clinical Research Center for Cancer, State Key Laboratory of Druggability Evaluation and Systematic Translational Medicine, Tianjin Medical University Cancer Institute and Hospital, Tianjin Medical University, Tianjin, China; 2 Department of Lung Cancer, Key Laboratory of Cancer Prevention and Therapy, National Clinical Research Center for Cancer, Tianjin’s Clinical Research Center for Cancer, Tianjin Medical University Cancer Institute & Hospital, Tianjin, China; Stanford University, UNITED STATES OF AMERICA

## Abstract

Lung cancer (LC) is the leading cause of cancer-related mortality worldwide, accounting for millions of deaths annually. Its major subtypes—lung squamous carcinoma (LUSC), lung adenocarcinoma, and small-cell LC—exhibit distinct risk factors and genetic susceptibilities, necessitating the use of subtype-specific biomarkers. Two-sample Mendelian randomization (MR) analyses were conducted using protein quantitative trait loci from the UK Biobank Pharma Proteomics Project and deCODE datasets. A robust analytical framework, including reverse MR, meta-analysis, summary-data-based MR tests, and colocalization, cisMR-cML, MR.CUE and phenotype scanning analyses were used to identify proteins associated with LC risk. We conducted a systematic review to contextualize our research findings. Follow-up analyses, including pathway enrichment, protein-protein interaction network analysis, and druggability evaluations, were used to explore the mechanisms and therapeutic potential of the identified proteins. Significant proteins were validated using population-level proteomic data from the UK Biobank (UKB). The results showed that twenty-five proteins were significantly associated with LC or its subtypes, including 15 novel findings. 60S ribosomal protein L14 (RPL14) and advanced glycosylation end-product-specific receptor (AGER) emerged as the strongest discovery, demonstrating consistent and significant associations across both MR and population-level analyses. RPL14 exhibited positive associations with overall LC risk (MR_meta: odds ratio [OR]: 2.012, 95% confidence interval [CI]: 1.297–3.119; UKB: OR: 1.509, 95% CI: 1.015–2.244). Similarly, AGER showed significant protective effects against LUSC risk (MR_meta: OR: 0.572, 95%CI: 0.368–0.889; UKB: OR: 0.366, 95% CI: 0.158–0.850). Pathway analysis revealed the involvement of these proteins in immune regulation and tumorigenesis. Among the 13 identified druggable targets, RPL14 and AGER showed therapeutic potential as approved or investigational drugs targeting these proteins. These findings offer new insights into the pathogenesis of LC and potential therapeutic targets.

## Author summary

Lung cancer is responsible for millions of deaths worldwide each year, creating a substantial health burden. Proteins, as the end products of gene expression and essential functional components in biological processes, serve as valuable biomarkers and therapeutic targets for complex diseases. However, different subtypes exhibit varying genetic susceptibilities, and the connections between a wide range of proteins and lung cancer remain unclear. Therefore, investigating the relationships between proteins and lung cancer, along with its subtypes, is crucial for uncovering insights into risk prediction and treatment. In our study, we explored the associations between thousands of proteins and lung cancer, identifying causal links for twenty-five proteins related to lung cancer risk or its subtypes. New associations in our findings were identified using systematic reviews. Based on population evidence, we highlighted the significance of the RPL14 and AGER in lung cancer risk. We also proposed that RPL14 and AGER may play roles in lung carcinogenesis and serve as therapeutic targets, supported by follow-up analyses. These findings hold important implications for both predicting lung cancer risk and developing innovative treatment strategies.

## Introduction

Lung cancer (LC) remains the predominant cause of cancer-related mortality globally, accounting for approximately 1.8 million fatalities in 2022 [1]. The most prevalent histological subtype is lung adenocarcinoma (LUAD, 45%), followed by lung squamous carcinoma (LUSC, 20%), small-cell LC (SCLC, 11%), and large-cell lung carcinoma (7%) [2]. The 5-year survival rates for patients with LC are relatively low, ranging from 10% to 20% in most countries [3–5]. Despite advancements in the screening and treatment of LC, early detection and targeted anticancer therapies remain the most promising modalities for enhancing long-term survival [6]. Exploring noninvasive biomarkers holds significant potential for understanding the LC etiology and developing tailored treatment strategies [7,8].

Proposed biomarkers span a wide range, including single nucleotide polymorphisms (SNPs), circulating tumor DNA methylation, microRNAs, metabolites, and proteins [9–15]. Proteins, as the end products of gene expression and primary functional components of biological processes, represent promising intermediate phenotypes for discovering novel mechanisms underlying major chronic diseases [16]. Previous observational research leveraging high-throughput proteomic technologies has identified several circulating proteins associated with LC risk across various pathological types [17,18]. However, potential residual confounding bias (e.g., arising from unmeasured or inaccurately measured confounders) and reverse causality in observational research limit the ability to draw causal inferences from these studies.

Mendelian randomization (MR) study is a powerful causal inference tool in epidemiological research, known as Mother Nature’s randomized clinical trial (RCT) [19]. It utilizes genetic variants strongly associated with exposure (anthropometric characteristics, behavior, circulating proteins/metabolites/genes, etc.) as instrumental variables (IVs) to infer causal associations between exposure and outcome. Because these genetic variants are acquired naturally and randomly at fertilization (meiosis), the associations revealed by MR are not subject to reverse causation and are unlikely to be affected by confounding factors, effectively addressing the limitations of observational studies [19]. LC has a significant genetic basis (heritability ~ 18%) and familial history (first-degree relative) confers a 50% risk elevation [20–22]. Consequently, MR has been widely adopted to investigate its etiology. Given subtype-specific variations in genetic susceptibility loci and key risk factors (e.g., smoking, particulate matter exposure, automobile exhaust), distinct circulating protein biomarkers likely exist across subtypes [23–25]. However, a systematic review of existing MR studies on circulating proteins and LC risk (S1 and S2 Tables) reveals limitations in sample size [26], proteome coverage [27], subtype-specific analyses [23], and real-world population validation. Future studies should address these gaps to strengthen causal inference.

This study combined data from two extensive proteomic GWAS studies and two large LC GWAS studies to explore the associations between circulating proteins and the risk of LC and its three subtypes. Using MR methods and multiple analytical approaches (meta-analysis, summary-data-based MR [SMR] tests, colocalization, cisMR-cML, MR.CUE, and phenotype scanning), we identified 25 significant proteins linked to LC or its subtypes, 15 of which were novel. Follow-up assessments further revealed the functions of these proteins, identifying 13 proteins (e.g., RPL14, AGER, PDE5A) as having therapeutic potential. In addition, individual-level data obtained from the UK Biobank (UKB) validated the associations between 60S ribosomal protein L14 (RPL14) and LC risk, as well as advanced glycosylation end-product-specific receptor (AGER) and LUSC risk.

## Methods

### Ethics statement

The original studies of GWAS data used in this study have been approved by relevant ethical review institutions and informed consent of participants. This study only analyzed publicly available summary-level statistical data, and therefore, no new ethical review board approval was required. UK Biobank received ethics approval from the Research Ethics Committee (REC reference for UK Biobank is 21/NW/0157). The UK Biobank data used in this study were under the approved application number 76092. All participants provided written informed consent, and the North West Multi-centre Research Ethics Committee approved the study.

**Fig 1:**
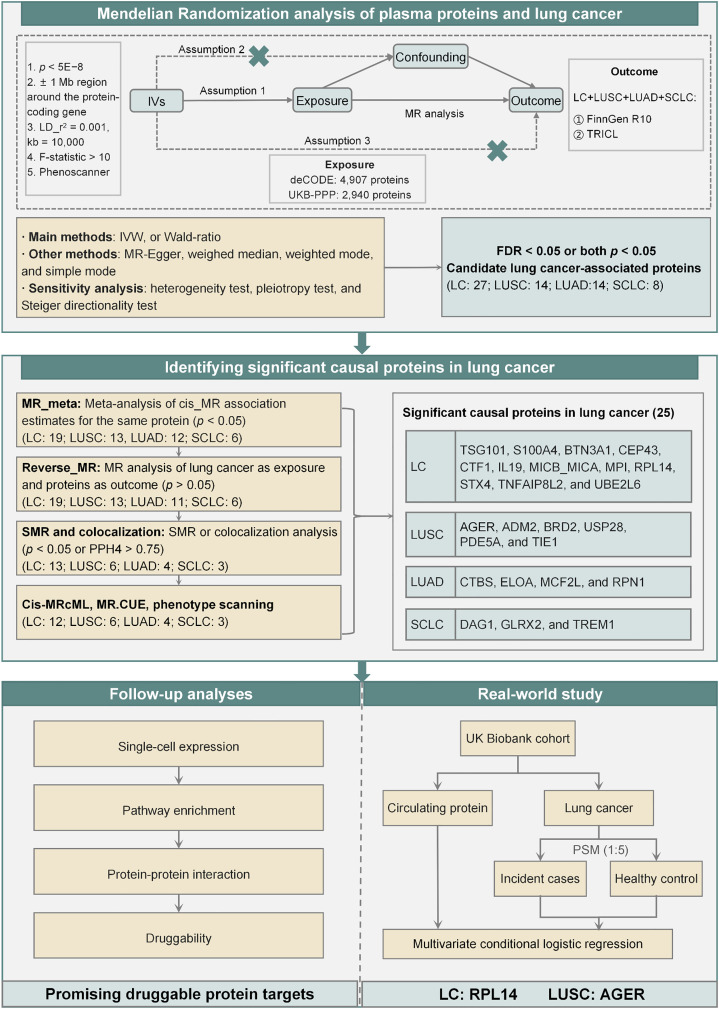
Flowchart with the study design. (LD: linkage disequilibrium; IVs: instrument variables; IVW: inverse variance weighted; MR: Mendelian randomization; LC: lung cancer; LUSC: lung squamous carcinoma; LUAD: lung adenocarcinoma; SCLC: small cell lung cancer; UKB-PPP: the UK Biobank Pharma Proteomics Project; SMR: summary-data-based MR test; PSM: propensity score matching).

Fig 1 shows the comprehensive study design. Using cis-protein quantitative trait locus (cis-pQTL) as genetic instrumental variables, we conducted two-sample MR analyses to identify candidate causal proteins associated with LC risk. Meta-analyses, reverse MR analyses, SMR tests, HEIDI tests, colocalization analyses, cisMR-cML, MR.CUE and phenotype scanning analyses were conducted to identify significant causal proteins linked to LC risk. For the significant proteins, our review helped identify novel associations. Furthermore, follow-up analyses were performed to investigate the potential mechanisms underlying the proteins involved in LC carcinogenesis and their druggability. Additionally, we used plasma proteomic data from the UK Biobank to search for evidence of associations between the prediagnostic circulating levels of significant proteins and LC risk.

### Data sources

Summary-level GWAS data for circulating proteins were obtained from two large cohorts: the UK Biobank Pharma Proteomics Project (UKB-PPP) and the deCODE study. The UKB-PPP study examined plasma proteins in 54,219 participants with an average age of 57 years and publicly available GWAS data for 2,940 (2,922 unique) proteins [28], as detailed in S3 Table. The deCODE study involved 35,559 Icelanders with a mean age of 55 years and measured the 4,667 unique plasma protein levels of 4,907 aptamers [29], as detailed in S4 Table. The two studies covered a total of 1,822 overlapping proteins.

The GWAS data for LC and its three subtypes (LUSC, LUAD, and SCLC) were obtained from the FinnGen R10 release data, with a sample size of more than 412,181 Finnish individuals and a median age of 63 years [30]. The other summary-level GWAS data of LC were obtained from GWAS Catalog datasets and carried out under the auspices of the Transdisciplinary Research in Cancer of the Lung (TRICL) study linked to the International Lung Cancer Consortium. A total of 29,266 patients with LC and 56,450 healthy controls were included, of whom 88% were over 50 years of age [31]. Table 1 provides an overview of the websites for the GWAS data sources used in this study.

**Table 1:** The GWAS data source used in this study.

Dataset	Trait	Sample size	Ncase	PMID	Website
UKB-PPP	2,940 proteins	54,219	–	37794186	https://www.synapse.org/#!Synapse:syn51364943/wiki/622119
deCODE	4,907 proteins	35,559	–	34857953	https://www.decode.com/summarydata/
FinnGen	LC	320,533	6,340	36653562	C3_BRONCHUS_LUNG_EXALLC (https://www.finngen.fi/en)
	LUSC	315,703	1,510	36653562	C3_NSCLC_SQUAM_EXALLC (https://www.finngen.fi/en)
	LUAD	315,783	1,590	36653562	C3_NSCLC_ADENO_EXALLC (https://www.finngen.fi/en)
	SCLC	314,910	717	36653562	C3_SCLC_EXALLC (https://www.finngen.fi/en)
TRICL	LC	85,716	29,266	28604730	GCST004748 (http://www.ebi.ac.uk/efo/EFO_0005543)
	LUSC	63,053	7,426	28604730	GCST004750 (http://www.ebi.ac.uk/efo/EFO_0000708)
	LUAD	66,756	11,273	28604730	GCST004744 (http://www.ebi.ac.uk/efo/EFO_0000571)
	SCLC	24,108	2,664	28604730	GCST004746 (http://www.ebi.ac.uk/efo/EFO_0000702)

LC: lung cancer; LUSC: lung squamous carcinoma; LUAD: lung adenocarcinoma; SCLC: small-cell lung cancer; UKB-PPP: the UK Biobank Pharma Proteomics Project; TRICL: the Transdisciplinary Research In Cancer of the Lung Research Team.

The population-level proteomic data were derived from the UK Biobank, which is a large prospective cohort study that collects information regarding demographics, body measurements, diet, lifestyle, health status, plasma markers, and genetic data from 500,000 participants aged 40–69 years recruited in 2006–2010 [32]. Proteomic data were provided as Normalized Protein eXpression (NPX) values on a log_2_ scale measured using an antibody-based Olink Explore 3072 PEA panel [28].

### Two-sample MR analyses to identify candidate proteins

We used univariate MR to determine the association of circulating proteins with LC using cis-pQTL (± 1Mb) as genetic IVs (detailed in S1 Text). When only one genetic variant was available, analysis was performed using the Wald ratio method. When two or more genetic variants were present, the analysis was performed using the inverse variance weighted (IVW) method of the random-effects model. Meanwhile, MR-Egger, weighted median, weighted mode, and simple mode were used for supplementary analyses [33]. Heterogeneity and pleiotropy tests were conducted using Cochran’s Q statistic and the MR-Egger intercept, respectively. Steiger directionality tests were used to detect the direction of MR association, the result shows “TRUE” means it is from exposure to outcome. The power of MR analyses was measured using the mRnd online tool. The Benjamini-Hochberg method was used to adjust for multiple tests.

Candidate LC-associated proteins were identified if they fulfilled any of the following conditions: (1) significantly associated with LC from any source (false discovery rate [FDR] < 0.05) or (2) associated with LC from both the FinnGen and TRICL databases (**p* *< 0.05).

### MR_meta, reverse_MR, SMR, colocalization, cisMR-cML, MR.CUE and phenotype scanning analyses to identify significant proteins

We identified significant proteins in four stages.

First, we performed a meta-analysis of the MR results for the candidate proteins and LC in the TRICL and FinnGen studies. A random-effects model was used when heterogeneity was observed, while a fixed-effects model was applied otherwise. Heterogeneity was defined as a *p* < 0.05 in the Q-test.

Second, we performed reverse MR analyses to avoid the effect of reverse causality. When **p* *< 0.05, it represents that the presence of LC can affect protein levels, indicating a possible reverse causality. The IV screening approach for LC is shown in S1 Text.

Third, SMR and colocalization analyses were conducted to validate the association between plasma proteins and LC (FinnGen) [34]. SMR analyses were performed using the default parameters (cis-wind = 2,000 kb, trans-wind = 1,000 kb, peqtl-trans = 5e-8, peqtl-other = 1e-5, thread-num = 10, and diff-freq-prop = 0.3). The HEIDI tests were used for sensitivity analyses to distinguish whether the causal relationships obtained were due to shared genetic variation rather than chain disequilibrium effects [34,35]. The significance of SMR analysis was defined as *p*_SMR_ < 0.05 and *p*_HEIDI_ > 0.01. Using the parameters of p_1 _= 1 × 10^-4^, p_2 _= 1 × 10^-4^, and p_12 _= 1 × 10^-5^, we conducted a colocalization analysis between plasma proteins and LC, and shared genetic variations were deemed to be present when the posterior probability of hypothesis 4 (PPH4) > 0.75 [23]. Proteins that satisfied the SMR or colocalization evidence would be processed for the fourth step of validation.

Fourth, to strengthen the robustness of causal inference and to correct for pleiotropic interference effects, we performed cisMR-cML and MR.CUE analyses of significant proteins and phenotype scanning analyses of IVs. In cisMR-cML analysis, we selected genetic variants based on cis-pQTL (±1Mb), using conditional and joint association analysis (called GCTA-COJO) to select SNPs [36,37]. Then, the cismr_cML_DP function is used to determine the association effects between proteins and LC. In MR.CUE analysis, using the independent reference panel data provided by Chen et al [38]. Preset pva_cutoff = 5E-8 to pick significant IVs, using the *ReadSummaryStat* function to match the three data sets (exposure, outcome, and panel data) and align effect sizes, where the shrinkage transformation parameter *lambad* = 0.85 for the linkage disequilibrium (LD) estimator is set [38]. The *MRCUE* function was used to estimate the association effect. Significance of any one of cisMR-cML, MR.CUE analyses were demonstrated by proving the robustness of the causal association. For phenotype scanning, we utilized SNP-phenotype association data from the GWAS Catalog (v1) to look at other relevant phenotypes for IV of the study proteins [39]. We excluded SNPs associated with known risk factors (e.g., smoking, asthma, chronic obstructive pulmonary disease [COPD]) from significant protein IVs (p ≤ 1E-5) and subsequently repeated the cis-MR analyses to confirm result stability. See more information in the S1 Text.

### Follow-up analyses

To gain deeper insights into the mechanism of action of the significant proteins and their druggability, we performed single-cell expression analysis, pathway enrichment analysis, protein-interaction (PPI) network analysis, and druggability assessments. Single-cell expression analysis was used to investigate the cell types in which the genes encoding the significant proteins were predominantly expressed in lung tissues using the Human Protein Atlas database [40]. Pathway enrichment analysis was carried out on the Metascape website, with the protein-coding genes of UKB-PPP and deCODE as background gene sets, with the following screening criteria: minimum overlap = 3, p-value threshold = 0.05, and minimum enrichment = 1.5 to identify pathways and processes enriched for significant proteins [41]. To clarify the interactions between the significant proteins, as well as between the significant proteins and established LC therapy targets, we queried the drug targets related to LC in DrugBank (https://go.drugbank.com/) and then discovered the interactions using the STRING database. Cytoscape software was used to map PPI plots. Finally, we searched four drug target-related databases: DrugBank [42], Therapeutic Target Database (TTD, https://go.drugbank.com/) [43], ChEMBL (https://www.ebi.ac.uk/chembl/) [44], and DGIdb (https://www.dgidb.org/) [45], to further assess whether the significant proteins could be potential therapeutic targets. We documented the details of the drug names and possible indications related to the significant proteins.

### Real-world study

S1 Fig and S1 Text present the study population screening flowchart and data processing. Using a nested case-control design, we performed propensity score matching (PSM) at a 1:5 ratio to match LC new cases (N = 322) with cancer-free controls (N = 1,610). The LC cases comprised LUSC (N = 47), LUAD (N = 103), and SCLC (N = 35). PSM was performed based on logistic regression-derived propensity scores. Matching covariates included age, sex, ethnicity, Townsend deprivation index (TDI), and family history of LC. Nearest neighbor matching was implemented with a caliper width of 0.1 standard deviations of the propensity score. Post-matching covariate balance was evaluated using standardized mean differences (SMD), with SMD < 0.1 indicating adequate balance. Multivariable conditional logistic regression models were used to assess associations between circulating proteins and lung cancer risk. Adjustment variables—determined based on scientific rationale and prior publications—included body mass index (BMI), smoking status, drinking status, physical activity, healthy diet, respiratory diseases, and blood glucose [46,47]. See S5 Table for variable definitions.

All participants provided written informed consent, and the North West Multi-centre Research Ethics Committee approved the study. The UK Biobank data used in this study were under the approved application number 76092.

## Results

### Identification of candidate LC-associated proteins

After screening and harmonizing with outcome data, the two studies covered a total of 879 overlapping proteins, with an additional 1,062 unique proteins provided by UKB-PPP and 792 unique proteins supplied by the deCODE study, resulting in a total of 2,733 unique predicted proteins analyzed. The F-statistics of the instrumental variables for these proteins were all higher than 10. The proportion of variance in exposure explained by a single genetic variant ranged from 0.0004 to 0.387, as outlined in S6 Table.

**Fig 2:**
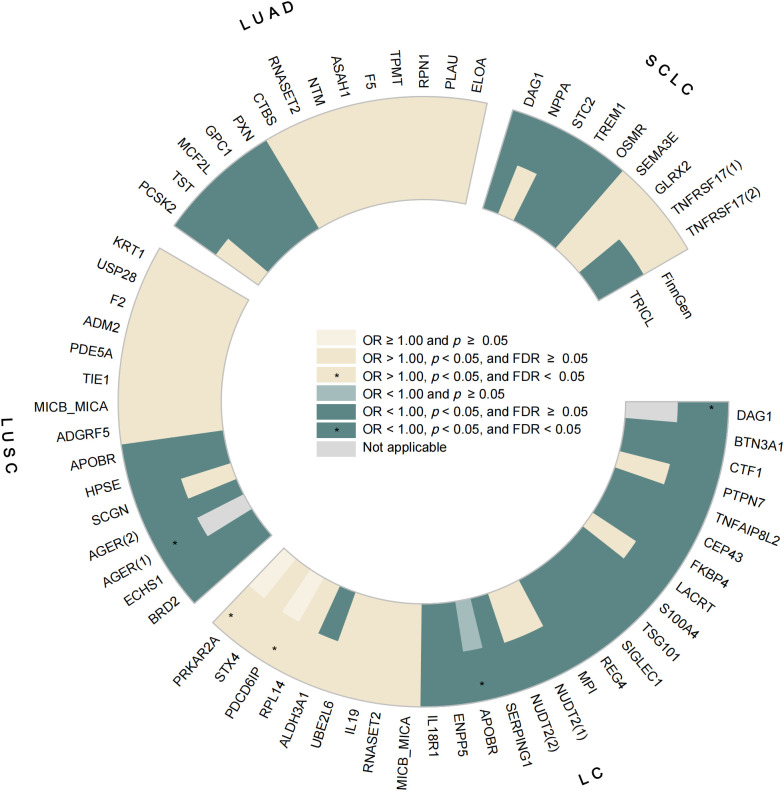
Candidate lung cancer-associated protein. (LC: lung cancer; LUSC: lung squamous carcinoma; LUAD: lung adenocarcinoma; SCLC: small cell lung cancer; Candidate lung cancer-associated proteins were defined as proteins that were significantly associated with lung cancer (FDR < 0.05); or with lung cancer from both sources (FinnGen and TRICL) (*p *< 0.05); OR: odds ratio; FDR: false discovery rate; (1): the protein source is the UKB-PPP project; (2): the protein source is the deCODE study; Not applicable: no SNPs were available for analysis after harmonization).

The complete results of the MR analyses are shown in S7–S22 Table. A total of 59 unique plasma proteins fulfilling the criteria for candidate protein screening were identified. Among these, 27 genetically predicted proteins were causally associated with LC risk, 14 with LUSC, 14 with LUAD, and eight with SCLC (Fig 2, S23 Table). Notably, four circulating proteins (APOBR, DAG1, MICB_MICA, and RNASET2) were causally linked to more than one LC subtype, while the remaining 55 proteins showed evidence of a primary causal association with only one LC subtype. Additionally, genetically predicted levels of AGER identified from both exposure databases were highlighted as candidate causal proteins, suggesting stronger evidence of association.

No evidence of heterogeneity or pleiotropy was observed (**p* *> 0.05). Steiger directionality tests confirmed that the direction of causality consistently pointed from proteins to LC risk. Most of these analyses achieved a statistical power of up to 80%, as detailed in S24 Table.

### Identification of significant LC-associated proteins

The meta-analyses’ results revealed that 48 genetically predicted proteins were associated with the risk of LC or its subtypes. Significant associations were observed between 19 proteins and overall LC risk, 13 proteins with LUSC, 12 proteins with LUAD, and six proteins with SCLC (S25 Table). Notably, two of these proteins were causally linked to an increased risk of two LC subtypes. Specifically, genetic liability to elevated MICB_MICA protein levels increased the risk of LC and LUSC, while genetically predicted high RNASET2 levels were associated with an increased risk of LC and LUAD. Of the remaining proteins, 26 showed negative associations with a specific LC subtype, while 20 demonstrated positive associations.

The number of IVs for overall LC, LUSC, LUAD, and SCLC from the FinnGen database was 7, 2, 1, and 4, respectively, while the number of IVs from the TRICL database was 14, 7, 10, and 2. All IVs exhibited F-statistics greater than 10, and the proportion of variance explained by a single genetic variant ranged from 0.004 to 0.046. Reverse MR analyses with LC as the exposure and protein levels as the outcome, suggested that protein PLAU may be affected by LUAD (S26 Table). To avoid the potential reverse causality effect, this protein was excluded from subsequent analyses.

Furthermore, the results of the SMR analyses indicated that 26 proteins were significantly associated with LC or its subtypes (**p* *< 0.05), with the direction of these associations aligning with the MR_meta evidence (S27 Table). Among them, 13 genetically predicted proteins were significantly causally associated with overall LC risk. Five proteins (MICB_MICA, IL19, UBE2L6, RPL14, and STX4) were associated with elevated risk, while eight (BTN3A1, CTF1, TNFAIP8L2, CEP43, S100A4, TSG101, MPI, and SERPING1) were linked to reduced risk. Six proteins were significantly causally associated with LUSC risk. Four proteins (TIE1, PDE5A, ADM2, and USP28) were causally linked to an increased risk, while two (BRD2 and AGER) were causally associated with reduced risk. In the case of LUAD, four proteins showed a significant causal link. Three (CTBS, RPN1, and ELOA) were linked to an increased risk of LUAD, whereas MCF2L was associated with a decreased risk. Finally, three proteins were significantly causally associated with SCLC risk: GLRX2 was linked to an increased risk, while TREM1 and DAG1 were identified as protective factors.

The HEIDI test indicated no heterogeneity in these associations (**p* *> 0.01). Notably, the colocalization analysis provided strong support for two associations: AGER with LUSC (PPH4 = 0.821) and CTBS with LUAD (PPH4 = 0.785), indicating a high probability of shared causal variability between protein levels and LC risk (S28 Table).

CisMR-cML and MR.CUE analyses revealed causal associations between 25 proteins and specific LC subtypes, supported by at least one method. However, the association of SERPING1 with overall LC risk may be influenced by pleiotropy and was not validated through either approach (S29 and S30 Tables). Phenotype scanning identified associations between rs204993 (AGER, ID = oid20756) and asthma, as well as rs117960683 (SERPING1, ID = 4479_14_SERPING1_C1_Esterase_Inhibitor) and daily smoking (S31 Table). After removing these SNPs, AGER maintained a significant protective association with LUSC risk, whereas the association between SERPING1 and LC risk (FinnGen) was no longer robust (S32 Table).

**Fig 3:**
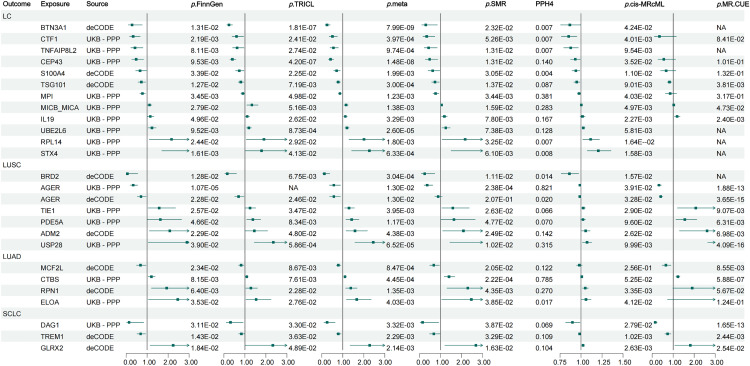
Forest plot of the significant lung cancer-associated proteins. (LC: lung cancer; LUSC: lung squamous carcinoma; LUAD: lung adenocarcinoma; SCLC: small cell lung cancer; UKB-PPP: the UK Biobank Pharma Proteomics Project; TRICL: the Transdisciplinary Research In Cancer of the Lung Research Team; SMR: summary-data-based MR test; PPH4: the posterior probability of hypothesis 4 in colocalization analysis; NA: not applicable, no SNPs were available for analysis after harmonization).

The summary of 25 significant protein-LC associations is presented in S33 Table and Fig 3. Compared with 15 MR studies on circulating proteins and LC risk identified through our systematic review (S34 and S35 Tables), 10 protein-LC associations were further validated (LC: IL19, MICB_MICA, TSG101, and CTF1; LUSC: PDE5A and TIE1; LUAD: MCF2L and RPN1; and SCLC: GLRX2 and TREM1), while the remaining 15 causal associations represent novel findings (LC: RPL14, STX4, UBE2L6, S100A4, BTN3A1, CEP43, MPI, and TNFAIP8L2; LUSC: ADM2, USP28, AGER, and BRD2; LUAD: CTBS and ELOA; and SCLC: DAG1).

### Follow-up analysis

#### Single-cell expression.

**Fig 4:**
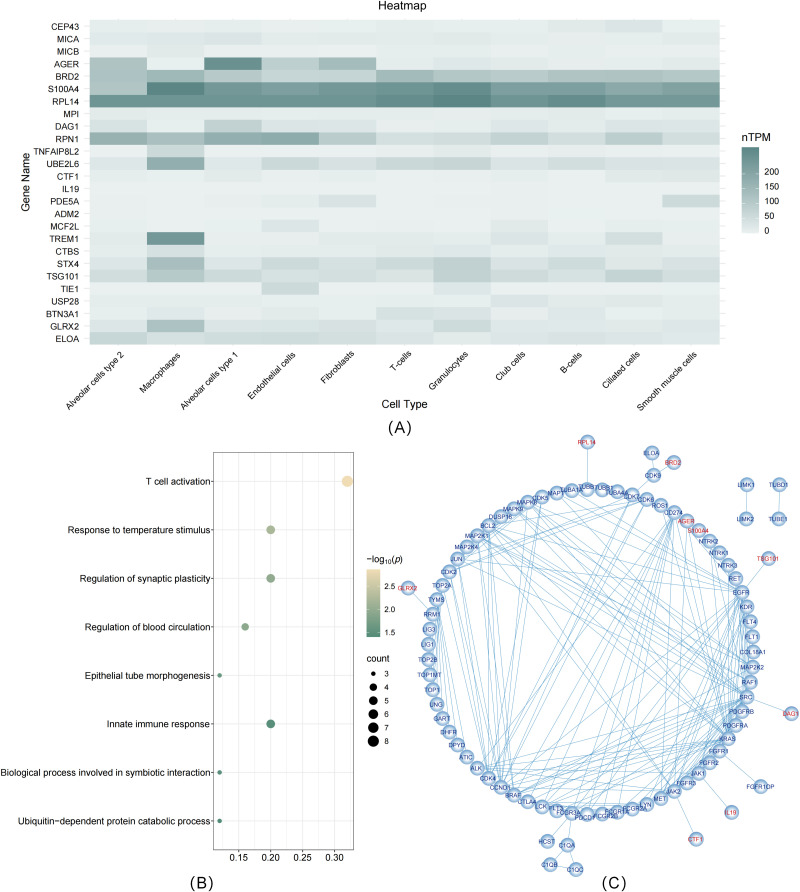
Follow-up analyses of significant lung cancer-associated proteins. (A: single-cell expression types of significant proteins; B: pathway enrichment analysis of significant proteins; C: Protein-Protein Interaction Networks of significant proteins; nTPM: Transcripts Per Million, all TPM values per sample were scaled to a sum of 1 million TPM).

These 25 significant protein-coding genes were primarily expressed in 11 distinct cell types in the lung, including alveolar cells (type 1/2), macrophages, endothelial cells, fibroblasts, T cells, granulocytes, club cells, B cells, ciliated cells, and smooth muscle cells (Fig 4A and S36 Table). Notably, BRD2, RPL14, and S100A4 were predominantly enriched in these cells, while AGER was particularly enriched in alveolar cells and fibroblasts.

#### Pathway enrichment.

Pathway enrichment analysis revealed that these proteins were primarily involved in pathways related to T cell activation, regulation of blood circulation, innate immune response, and other biological activities. These findings indicate that these proteins play crucial roles in biological processes such as immune system regulation and blood circulation control (Fig 4B and S37 Table).

#### PPI network.

Using the DrugBank database, we retrieved 88 LC-related drug targets (S38 Table). By combining these targets with the 25 significant proteins, we performed a PPI network analysis. Nine proteins (AGER, S100A4, TSG101, RPL14, DAG1, IL19, CTF1, GLRX2, and BRD2) interacted with known LC targets, such as TSG101 with EGFR. Additionally, AGER interacted with S100A4, suggesting involvement in inflammatory and immune processes (Fig 4C).

#### Druggability evaluation.

A search of four drug target databases identified 13 proteins with either investigational or approved drugs (S39 Table). Among these, PDE5A has been explored as a potential therapeutic target for LC. The drug Exisulind, targeting PDE5A, has potential applications in treating LC, colorectal adenomatous polyposis, and colon polyps. The remaining 12 proteins are also potential therapeutic targets and are currently associated with other diseases. Examples of approved drugs include AGER, targeted by Oxytocin (used for preeclampsia) and Vitamin B12 (used for anemia caused by B12 deficiency); BRD2, targeted by Triazolam (a sedative for insomnia) and Alprazolam (for anxiety disorders and epilepsy); CEP43 targeted by Brivanib, investigated for breast cancer and liver cancer; S100A4 targeted by Sp-Met-1, explored for melanoma, colorectal cancer, and breast cancer; RPL14 targeted by Artenimol, used for treating uncomplicated plasmodium falciparum infections.

### Real-world study

**Fig 5:**
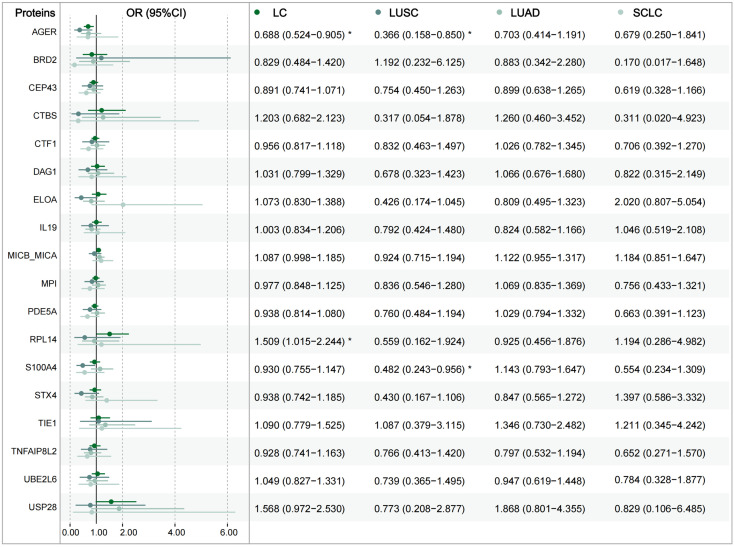
Association of significant proteins with lung cancer in UK Biobank Cohort. (The graph only shows the results of protein-lung cancer associations, which are detailed in S42 Table. LC: lung cancer; LUSC: lung squamous carcinoma; LUAD: lung adenocarcinoma; SCLC: small cell lung cancer; OR: odds ratio; CI: confidence interval).

The results of the balance assessment after matching and the baseline characteristics of the population are provided in S40 and S41 Tables, respectively. Multivariable logistic regression analysis identified 277 proteins significantly associated with overall LC risk (109 protective/168 risk), 212 with LUSC (171 protective/41 risk), 118 with LUAD (65 protective/53 risk), and 169 with SCLC (103 protective/66 risk). Detailed information is provided in S42 Tables. Notably, RPL14 significantly increased overall LC risk (odds ratio [OR] = 1.509, 95% confidence interval [CI] = 1.105–2.244), while AGER reduced LUSC risk (OR = 0.366, 95%CI = 0.158–0.850), consistent with MR results (Fig 5).

## Discussion

This study conducted comprehensive MR analyses to explore causal associations between a total of 2,733 unique genetically predictive proteins from the UKB-PPP and deCODE study with LC and its three subtypes. Through meta-analyses, SMR tests, and colocalization analyses, cisMR-cML, MR.CUE and phenotype scanning analyses, we identified 12, 6, 4, and 3 genetically predicted proteins causally associated with susceptibility to overall LC, LUSC, LUAD, and SCLC, respectively. Our systematic review of previous MR studies on proteins and the risk of LC suggested that 15 of these protein-LC associations were considered newly identified. Additionally, 13 proteins—including six novel proteins (AGER, RPL14, S100A4, CEP43, MPI, and BRD2)—were identified as potential therapeutic targets. Further validation using population-level proteomic data from the UK Biobank confirmed a significant association between RPL14 and an increased risk of LC, as well as protein AGER and a reduced risk of LUSC.

The genetic architecture of different LC subtypes exhibits significant heterogeneity, with a growing body of research identifying novel subtype-specific genetic variants [31]. This heterogeneity significantly influences treatment strategies for distinct LC subtypes, particularly in the realm of pharmacogenomics [48]. Proteins, as the most widely utilized biomarkers for disease diagnosis, prognosis, and drug development, make the investigation of subtype-specific associated proteins imperative. However, most previous studies have predominantly focused on overall LC rather than comprehensively addressing individual subtypes. Thus, among the 10 protein-LC associations from our study that aligned with previous findings, the majority were linked to overall LC risk, including IL19, MICB_MICA, TSG101, and CTF1.

Among the novel potential therapeutic targets for LC identified in our study, the association of protein RPL14 with overall LC and AGER with LUSC has been robustly demonstrated across genetic and real-world population studies. RPL14 is a member of the L14E ribosomal protein family. The protein consists of five introns and six exons and is located on chromosome 3p21.3 [49]. At the genetic level, the heterozygosity of the *RPL14* locus is 68%, while it is only 25% in non-SCLC (NSCLC) cell lines. This suggests that RPL14 may play a potential role in LC development due to the loss of heterozygosity in the mechanism of LC [50]. Additionally, RPL14 plays a role in the occurrence and progression of other cancers, such as esophageal squamous carcinoma [51], hepatocellular carcinoma [52], and cervical cancer [53]. AGER is a transmembrane pattern recognition receptor in the immunoglobulin superfamily [54]. It is predominantly expressed in the lungs, where its normal expression level plays a critical role in maintaining alveolar type 1 (AT1) cell differentiation, adhesion, spreading, and apoptosis. Inhibition of AGER expression in alveolar cells or its downregulation during malignant transformation in LC cells enhances migratory, invasive, and proliferative properties, thereby promoting LC development [54,55]. In vitro studies demonstrated that co-culturing AGER-depleted human LC cells (H358) with lung fibroblasts (WI38) significantly enhanced cancer cell proliferation [56]. Clinically, reduced AGER protein expression in LUSC correlates with poor prognosis, while higher levels are associated with longer survival [57,58]. Additionally, AGER interacts with multiple endogenous ligands (e.g., HMGB1, S100 proteins, calreticulin) implicated in metastasis and disease progression within the LC microenvironment [59]. PPI analysis further revealed that AGER interacts with S100A4, and previous evidence suggests that S100A4 inhibits autophagy in an AGER-dependent manner, thereby influencing LC development. Knol et al. used data-independent acquisition mass spectrometry to report The Pan-Cancer Proteome Atlas (TPCPA) and highlighted AGER as a key LC biomarker [60]. We also observed a significant inverse association between AGER and overall LC risk in the UKB cohort (OR = 0.688). Given its mechanistic involvement and significant protective association with LC and LUSC, AGER is a promising target in LC research and therapeutic development.

Protein S100-A4 (S100A4) was associated with a reduced risk of overall LC in the MR analysis and a decreased risk of LUSC in the population analysis, aligning with Smith-Byrne et al.’s reported protective effect (OR = 0.814) [61]. While S100A4 is a well-established epithelial-mesenchymal transition marker implicated in metastasis across multiple malignancies [62,63], emerging evidence reveals its complex, context-dependent roles in cancer biology. On the one hand, S100A4 promotes tumor progression by up-regulating mitochondrial complex I subunit NADH dehydrogenase (ubiquinone) Fe-S protein 2 to enhance invasion and metabolic reprogramming [64]. Clinically, high S100A4 expression is associated with poor differentiation and poor prognosis of NSCLC [65–67]. On the other hand, S100A4 induction has also been shown to reduce motility and invasiveness, for example, in squamous cell carcinoma [68]. Upregulation of S100A4 by P53 mutant (H1299) contributes to the restoration of the tumor suppressor activity of plakoglobin, a tumor/metastasis suppressor [69]. Downregulation of S100A4 in astrocytes increased their migration capacity in vitro [68]. These seemingly contradictory observations suggest a biphasic role for S100A4 in lung carcinogenesis - acting as a tumor progression inhibitor during early disease stages while facilitating metastatic dissemination in advanced tumors [70]. Mannose-6-phosphate (MPI) was also found to be associated with a reduced risk of LC in our study. Multiple types of tumor cells exhibit enhanced glucose uptake characteristics, MPI could influence neoplastic growth by concurrently inhibiting critical metabolic pathways - glycolysis, Tricarboxylic acid cycle, and glycoconjugate biosynthesis [71]. Notably, MPI has been shown to potentiate therapeutic responses in acute myeloid leukemia, enhancing cellular sensitivity to both conventional cytarabine chemotherapy and targeted FLT3 inhibitors [71]. Furthermore, Luan et al. discovered that MPI is associated with a reduced risk of NSCLC, suggesting its potential tumor-suppressive role in pulmonary malignancies [72]. Further studies are needed to precisely delineate their context-specific mechanisms in LC, which may provide novel insights for targeted therapeutic strategies.

In addition, MR evidence from this study also supports the associations of Interleukin 19 (IL19) and Plasma protease C1 inhibitor (SERPING1) with LC. IL19, a member of the IL10 cytokine family, is primarily produced by monocytes and exerts its effects via IL-20R1/IL-20R2 chain complexes [73]. Our study demonstrated a significant association between elevated IL19 levels and an increased risk of LC, consistent with the findings reported by Zhang [74] and Fang [75]. Furthermore, in vitro assays indicated that elevated IL19 levels induce apoptosis in lung epithelial cells and promote neutrophil chemotaxis [76]. Other studies have also linked high IL19 expression to a poorer prognosis in interstitial lung disease among patients with SCLC [77]. Additionally, elevated IL19 levels have been implicated in bone metastasis from LC and the progression of other respiratory diseases, such as COPD and pulmonary fibrosis [78–80]. SERPING1, a member of the serine protease inhibitor superfamily, plays a key role in regulating inflammation. Its deficiency can result in angioedema [81]. In the current MR study, protein SERPING1 was consistently and significantly negatively associated with overall LC risk, aligning with findings from previous MR studies [82]. The analysis based on TCGA data indicated that SERPING1 expression was downregulated in LUAD [83]. Supporting this, one study reported that increased SERPING1 levels are associated with a poor prognosis in LUSC [84]. The role of SERPING1 in LC remains ambiguous, as there is a paucity of studies examining its relationship with specific subtypes. Additional research is necessary to elucidate the role of SERPING1 as either a protective or risk factor and to identify its potential mechanisms of action in LC pathogenesis.

Ubiquitin carboxyl-terminal hydrolase 28 (USP28), a catalytically active deubiquitinating enzyme, regulates key biological processes such as cell proliferation, DNA damage repair, apoptosis, and tumorigenesis [85]. Previous studies have shown that USP28 modulates the SREBP2 and mevalonate pathways, promoting tumor growth in LUSC [86]. USP28 has been implicated in the oncogenic transformation of respiratory cells, and its overexpression is associated with LUSC progression. Our findings are consistent with these studies, supporting the conclusion that USP28 contributes to increased LC risk, particularly in LUSC [87,88]. This study also identified several proteins that are significantly associated with LUAD and SCLC. Among these, the triggering receptor expressed on myeloid cells 1 (TREM1) was significantly associated with a decreased risk of SCLC. This finding was corroborated by Song et al., who also reported the protective role of TREM1 in SCLC [89]. TREM1 is a myeloid cell surface receptor involved in the tumor microenvironment, where it amplifies the inflammatory response and exhibits antitumor effects. Nie et al. demonstrated the potential therapeutic applications of TREM1 by developing a multichain DAP12/TREM1 CAR (DT CAR) targeting DLL3, which showed considerable antitumor effects in SCLC cells [90]. Despite these promising findings, the role of TREM1 in SCLC remains unclear. Further biological and mechanistic studies are required to elucidate its functions and potential as a therapeutic target for SCLC.

This study has the following strengths. First, we performed tens of thousands of causal association estimates based on two large-scale proteomics data sources and two LC data sources, selecting proteins with the highest level of evidence. Second, the newly published UKB-PPP project reported that 85% of its principal genetic associations were novel discoveries, thereby substantially expanding the potential for MR analyses to identify innovative protein biomarkers associated with LC pathogenesis. In addition, the large sample population protein data of UKB-PPP ensures temporal causality while providing detailed histopathological subtype information, significantly increasing the validity of our real-world evidence. Furthermore, this study incorporated multiple LC subtypes in the analysis and, based on an evidence grading system, highlighted the proteins with the strongest causal associations for each subtype. In addition, we conducted cisMR-cML and MR.CUE analyses effectively avoided the effect of pleiotropy. Finally, by combining genetic (MR, SMR, and colocalization) and population evidence (UK Biobank), we identified novel LC-associated proteins, enhancing the etiological network of LC.

This study has several limitations. First, European-focused GWAS data limited sex/age-stratified analyses and cross-ethnic generalizability. Second, given the analysis of thousands of plasma proteins, multiple testing correction may increase false negative rates [91]. To address this, we implemented a two-tiered screening strategy for primary protein selection: (1) FDR < 0.05 threshold, and (2) requiring statistically significant associations (*p* < 0.05) in two independent databases. This dual-validation approach maintains statistical rigor while effectively balancing false-positive and false-negative risks, and also identifies more potential proteins. Third, in screening primary candidate proteins, the direction of effect sizes is inconsistent between the two datasets, which may potentially be affected by factors such as the geographic background of the study population, the gender ratio, and differences in GWAS research methods. Fourth, the meta-analysis used the same exposures, which may cause some error in the results, but a series of subsequent analyses, such as SMR and colocalization, were carried out to minimize their effect on the results and ensure their reliability. Fifth, we identified subtype-specific proteins whose potential association with overall LC risk cannot be entirely excluded given shared genetic architecture. Their clinical interpretation thus requires larger studies or integrated evidence to clarify the underlying mechanisms. Sixth, although mean imputation in the UK Biobank cohort may lead to slight effect size attenuation through reduced variance, the impact is considered limited statistically, given the low average missing rate (~11%) in the protein data. Seventh, when enrichment analyses were performed, multiple corrections were not used because the small set of genes tested made it difficult to detect significant enrichment results. Finally, while this evidence strengthens our understanding of potential causal associations, these findings should be interpreted cautiously and further validated through experimental studies and large-scale population-based investigations.

## Supporting information

S1 Text Supplementary Methods. (S1_Text.DOCX)

S1 PRISMA Checklist (S1_PRISMA_Checklist.DOCX)

S1 STROBE MR Checklist (DOCX)

S1 Fig Flowchart of study population selection in UK Biobank. (LC: lung cancer; LUSC: lung squamous carcinoma; LUAD: lung adenocarcinoma; SCLC: small cell lung cancer; BMI: body mass index; TDI: Thompson Deprivation Index).
(S1_Fig.DOCX)

S1 Table Systematic review of Mendelian randomization studies on proteomics and lung cancer. (S1_Table.XLSX)

S2 Table Reasons for exclusion from the review literature on Mendelian randomization of circulating proteins and lung cancer (updated to August 28, 2024). (S2_Table.XLSX)

S3 Table Protein information for the UKB-PPP project. (S3_Table.XLSX)

S4 Table Protein information for the deCODE study. (S4_Table.XLSX)

S5 Table Definitions of adjustment factors and outcomes. (S5_Table.XLSX)

S6 Table Instrument variables of plasma protein in cis-MR analysis (after harmonization, F-statistics > 10). (S6_Table.XLSX)

S7 Table The Mendelian randomization results for plasma proteins (UKB-PPP) on lung cancer (FinnGen). (S7_Table.XLSX)

S8 Table The Mendelian randomization results for plasma proteins (UKB-PPP) and lung squamous carcinoma (FinnGen). (S8_Table.XLSX)

S9 Table The Mendelian randomization results for plasma proteins (UKB-PPP) and lung adenocarcinoma (FinnGen). (S9_Table.XLSX)

S10 Table The Mendelian randomization results for plasma proteins (UKB-PPP) and small-cell lung cancer (FinnGen). (S10_Table.XLSX)

S11 Table The Mendelian randomization results for plasma proteins (deCODE) and lung cancer (FinnGen). (S11_Table.XLSX)

S12 Table The Mendelian randomization results for plasma proteins (deCODE) and lung squamous carcinoma (FinnGen). (S12_Table.XLSX)

S13 Table The Mendelian randomization results for plasma proteins (deCODE) and lung adenocarcinoma (FinnGen). (S13_Table.XLSX)

S14 Table The Mendelian randomization results for plasma proteins (deCODE) and small-cell lung cancer (FinnGen). (S14_Table.XLSX)

S15 Table The Mendelian randomization results for plasma proteins (UKB-PPP) and lung cancer (TRICL). (S15_Table.XLSX)

S16 Table The Mendelian randomization results for plasma proteins (UKB-PPP) and lung squamous carcinoma (TRICL). (S16_Table.XLSX)

S17 Table The Mendelian randomization results for plasma proteins (UKB-PPP) and lung adenocarcinoma (TRICL). (S17_Table.XLSX)

S18 Table The Mendelian randomization results for plasma proteins (UKB-PPP) and small-cell lung cancer (TRICL). (S18_Table.XLSX)

S19 Table The Mendelian randomization results for plasma proteins (deCODE) and lung cancer (TRICL). (S19_Table.XLSX)

S20 Table The Mendelian randomization results for plasma proteins (deCODE) and lung squamous carcinoma (TRICL). (S20_Table.XLSX)

S21 Table The Mendelian randomization results for plasma proteins (deCODE) and lung adenocarcinoma (TRICL). (S21_Table.XLSX)

S22 Table The Mendelian randomization results for plasma proteins (deCODE) and small-cell lung cancer (TRICL). (S22_Table.XLSX)

S23 Table The summary results of Mendelian randomization analyses for candidate lung cancer-associated proteins. (S23_Table.XLSX)

S24 Table Statistical power, heterogeneity, pleiotropy, and Steiger directionality tests of Mendelian randomization analyses. (S24_Table.XLSX)

S25 Table The meta-analysis of the Mendelian randomization results. (S25_Table.XLSX)

S26 Table The Mendelian randomization results for lung cancer and plasma proteins. (S26_Table.XLSX)

S27 Table The results of SMR and HEIDI tests between plasma proteins and lung cancer. (S27_Table.XLSX)

S28 Table The colocalization analyses between plasma proteins and lung cancer. (S28_Table.XLSX)

S29 Table The results of cisMR-cML analysis between plasma proteins and lung cancer. (XLSX)

S30 Table The results of MR.CUE analysis between plasma proteins and lung cancer. (S30_Table.XLSX)

S31 Table The results of phenotype scanning. (S31_Table.XLSX)

S32 Table Cis-MR results of protein oid20756 (AGER, removal of rs204993) and 4479_14_SERPING1_C1_Esterase_Inhibitor (SERPING1, removal of rs117960683) with lung cancer risk. (S32_Table.XLSX)

S33 Table The summary results of 25 significant lung cancer-associated proteins. (S33_Table.XLSX)

S34 Table Systematic review of Mendelian randomization studies on proteomics and lung cancer. (S34_Table.XLSX)

S35 Table Summary information from previous Mendelian randomization studies. (S35_Table.XLSX)

S36 Table Single-cell type-specific expression analysis. (S36_Table.XLSX)

S37 Table The pathway of significant lung cancer-associated proteins enrichment. (S37_Table.XLSX)

S38 Table Drug targets in DrugBank for lung cancer treatment. (S38_Table.XLSX)

S39 Table Druggability evaluation of significant proteins. (S39_Table.XLSX)

S40 Table Propensity-matched equilibrium test for the UK biobank cohort. (S40_Table.XLSX)

S41 Table Baseline characteristics of lung cancer cases and matched controls after propensity score matching. (S41_Table.XLSX)

S42 Table Multivariate conditional logistic regression results of circulating proteins and lung cancer in the UK Biobank cohort. (S42_Table.XLSX)

## Abbreviations

LC: lung cancer
LUAD: lung adenocarcinoma
LUSC: lung squamous carcinoma
SCLC: small-cell lung cancer
SNPs: single nucleotide polymorphisms
MR: mendelian randomization
RCT: randomized clinical trial
IVs: instrumental variables
SMR: summary-data-based Mendelian randomization
UKB: UK Biobank
RPL14: 60s ribosomal protein l14
AGER: advanced glycosylation end-product-specific receptor
cis-pQTL: cis-protein quantitative trait locus
UKB-PPP: UK Biobank Pharma Proteomics Project
TRICL: Transdisciplinary Research In Cancer of the Lung
IVW: inverse variance weighted
PPH4: the posterior probability of hypothesis 4 in colocalization analysis
FDR: false discovery rate
LD: linkage disequilibrium
COPD: chronic obstructive pulmonary disease
PPI: protein-protein interaction
TTD: therapeutic target database
PSM: propensity score matching
TDI: Townsend deprivation index
SMD: standardized mean differences
BMI: body mass index
OR: odds ratio
CI: confidence interval
NSCLC: non-small-cell lung cancer
AT1: alveolar type 1
TPCPA: The Pan-Cancer Proteome Atlas
S100A4: protein S100-A4
MPI: mannose-6-phosphate
IL19: Interleukin 19
SERPING1: Plasma protease C1 inhibitor
USP28: Ubiquitin carboxyl-terminal hydrolase 28
TREM1: the triggering receptor expressed on myeloid cells 1
