# Supplementary material for: Circulating proteins associated with histological subtypes of lung cancer from genetic and population-based perspectives
Source: PLoS Genet. 2025 Aug 25;21(8):e1011821. doi: 10.1371/journal.pgen.1011821 (PMC12377608; doi:10.1371/journal.pgen.1011821)
Supplement: S1 Text — (S1_Text.DOCX) [file pgen.1011821.s001.docx]

**Supplementary Methods**

**Systematic review of Mendelian randomization (MR) studies**

The present review followed the Preferred Reporting Items for Systematic Reviews and Meta-Analysis (PRISMA) statement (**S1 PRISMA Checklist**)^[1]^. The detailed search strategy and literature scheduling details were described in **S1 and S2** **Tables**. Two investigators (ZYL and GJS) independently assessed the titles, abstracts, and full texts of all retrieved papers. Any discrepancies were addressed via discussion. This study concentrated on studies that employed MR analyses to examine the correlation between circulating proteins and lung cancer (LC). It excluded (1) non-original articles, such as reviews, conference abstracts, editorials, commentaries, correspondences, opinions, corrections, and study proposals; (2) studies that did not provide adequate original data, such as effect size (OR, odds ratio) and 95% confidence intervals (CIs) for the examined association; (3) duplicate publications, and (4) less than three circulating proteins (exposure).

We obtained the following information from the qualifying research using a predetermined Excel template: PMID, publication year, first author's name, exposure data source, sample type, proteomic approach (es), No. of protein quantitative trait locus (pQTLs), No. of proteins, outcome source, outcome, type of instrumental variable, study design, and effect metric (OR and 95%CI) for the exposure-LC and subtype association from the primary analysis. We collected the positive association outcomes (*p* < 0.05) in these studies. All data in each study were collected by ZYL and GJS on September 4, 2024.

**cis-MR analysis**

Instrumental variables (IVs) selection

We strictly followed the three basic assumptions of MR analysis to screen for the IVs for proteins: (1) the genetic variants were strongly associated with exposure (proteins). (2) The genetic variants affected the outcome (LC) risk only through exposure, not the confounders. (3) The genetic variants could not be directly associated with the outcome. First, cis-protein quantitative trait loci (cis-pQTL) with a genome-wide association threshold of *p* < 5E−8 were retained. Cis-pQTL were defined as genetically variable loci within ± 1 Mb of a protein-coding gene^[2]^. Second, to avoid the linkage disequilibrium (LD) effect, we set the condition of LD r^2^ < 0.001 and clumping size = 10,000kb for screening. Strong IVs with F-statistic greater than 10 were retained: R^2^ = 2 × EAF × (1 − EAF) × BETA^2^; F-statistic = R^2^ × (N − 2) / (1 − R^2^)^[3]^. Lastly, we queried IVs using the Phenoscanner online tool (http://www.phenoscanner.medschl.cam.ac.uk) to remove genetic variants directly associated with LC^[4]^. Six single nucleotide polymorphisms (SNPs: rs11751024, rs111791428, rs184716813, rs116350534, rs13191296, rs713875) directly associated with LC or its subtypes will be excluded on the basis of *p* < 1E-5. The STROBE-MR checklist was viewed in **S2 STROBE MR Checklist**.

Power calculations of MR analyses

We used the mRnd online tool (https://shiny.cnsgenomics.com/mRnd/) to calculate the statistical power of MR analyses^[5]^. It needs the following information: proportion of variance explained, OR, the proportion of cases in the study, sample size, and Type-I error rate (α = 0.05).

Reverse MR analyses

The screening of IVs for LC was performed similarly to the screening of IVs for proteins described above. Firstly, SNPs with strong association with LC (p < 5E-8), secondly, removing SNPs with LD (LD r^2^ < 0.001 and clumping size = 10,000kb), thirdly, screening SNPs with F-statistics higher than 10. The main methods for MR analysis were the inverse variance weighted (IVW) method or the Wald ratio method, and the complementary methods were MR-Egger, weighted median, weighted mode, and simple mode^[6]^. Sensitivity analyses, including heterogeneity and pleiotropy tests, were conducted using Cochran’s Q statistic and the MR-Egger intercept, respectively. Steiger directionality tests were used to detect the direction of MR association; the result shows “TRUE” means it is from exposure (LC) to outcome (proteins).

Colocalization analysis

Colocalization analysis only evaluates the probability of being affected by the same genetic variation between two traits rather than the direction of causal association, which is frequently regarded as a complementary analysis to MR. The results of the colocalization analysis can be viewed as supporting evidence of an association when the MR analysis indicates a significant association and the posterior probability of the colocalization analysis is higher than 0.7. This suggests that the genetic mechanisms underlying exposure and outcome may overlap^[7]^. The colocalization analysis was conducted utilizing the R package "coloc", which offers posterior probability for five scenarios concerning the likelihood that a single variable is shared by two traits: PPH0: unrelated to both proteins and LC; PPH1:only related to protein; PPH2: only related to LC; PPH3: related to protein and LC, but with different causal variants; PPH4: related to both protein and LC and with the same causal variants^[8]^. Using the default parameters of p_1_ = 1×10^-4^, p_2_ = 1×10^-4^, and p_12_ = 1×10^-5^, we conducted a colocalization analysis between plasma proteins and LC (FinnGen), and shared genetic variations were deemed to be present when PPH4 > 0.75^[9]^.

CisMR-cML analyses

The cisMR-cML method is used to estimate the effect of an exposure (e.g., gene or protein) on an outcome using publicly available summary genome-wide association study data where invalid instrumental variables may be present^[10]^. In cisMR-cML analysis, we selected genetic variants based on cis-pQTL (±1Mb), using conditional and joint association analysis (called GCTA-COJO) to select SNPS with marginal association with exposure (p < 5E-8), and excluded variants with high collinearity (r² ≥ 0.9) using the “--cojo-collinear 0.9” parameter; for some of the strongly collinearity proteins, the threshold was progressively adjusted to r² ≥ 0.8^[10, 11]^. Set a random seed (123), use 5 random starts, maximum number of iterations maxit = 200, number of perturbations num_pert = 100.

MR.CUE analyses

MR.CUE (MR with Correlated horizontal pleiotropy Unraveling shared Etiology and confounding) is a method to determine causal effects by accounting for correlated and uncorrelated horizontal pleiotropic effects^[12]^. In MR.CUE analysis, using the independent reference panel data provided by Chen et al^[12]^. Preset pva_cutoff = 5E-8 to pick significant IVs, using the *ReadSummaryStat* function to match the three data sets (exposure, outcome, and panel data) and align effect sizes, where the shrinkage transformation parameter lambad = 0.85 for the LD estimator is set. After which the random seed (50) is preset; rho = 0; L = length(F4gammah); opt = list (agm = 0, bgm = 0, atau1 = 0, btau1 = 0, atau2 = 0, btau2 = 0, a = 2, b = L, CluDes = “PropMajor”, maxIter = 1000, thin = 10, burnin = 500), and the MRCUE function was used to fit the MR.CUE analysis^[12]^.

**Follow-up analyses**

The Human Protein Atlas (HPA; https://www.proteinatlas.org/) database, based on 31 different single-cell transcriptome datasets, provides transcripts per million protein-coding genes (pTPM) for 85 cell types from 31 healthy tissues for download^[13]^. Single-cell transcriptome data from this database were utilized to investigate the cell types in which the genes encoding the focus proteins were mainly expressed in lung tissues, and the results are presented in the form of a heat map. To understand protein functions and pathways, the Metascape online website (https://metascape.org/) was utilized for pathway enrichment and bioprocess annotation of focus proteins. It provides a simpler workflow and incorporates several authoritative data sources, including GO, KEGG, UniProt, and other databases^[14]^. In this study, the conditions were set: screening criteria min overlap = 3, p-value = 0.05, min enrichment = 1.5, and bubble plots were used to display the results. The STRING database (https://string-db.org/) systematically collects and integrates protein-protein interactions, including physical interactions and functional associations^[15]^. To clarify the interactions between the significant proteins and whether there are any connections between the significant proteins and established targets for LC treatment. We first queried the drug targets related to lung cancer in DrugBank (https://go.drugbank.com/) and then combined the focus proteins to discover their interactions using the STRING database. The relevant parameters are set as follows: high confidence (0.700), false discovery rate (FDR) stringency (0.05). Next, we used Cytoscape software to map protein-protein interaction (PPI) after downloading the relevant association data^[16]^.

**Real-World Study**

Prior to processing, 850μl aliquots of EDTA plasma from the participants in the UK Biobank remained in a -80°C freezer. The Olink technology employs the Proximity Extension Assay to measure the expression level of proteins, where a matched pair of antibodies labeled with unique complementary oligonucleotides (proximity probes) binds to their target proteins in a sample. Antibodies targeting 2,923 unique proteins are distributed across eight 384-plex panels, which focus on inflammation, oncology, cardiometabolic, and neurological proteins^[17]^. The Olink proteome data were provided with Normalized Protein eXpression (NPX) values on a log2 scale. The participant's baseline time was the date of attending the assessment center (Field_id = 53). For study subjects for whom plasma protein testing was performed, only baseline plasma protein data with ins_index = 0 were retained; non-baseline plasma protein data with ins_index ≠ 0 were set as missing values and excluded along with individuals who were not tested for plasma protein. This study’s outcome was incident lung cancer, identified through linkage with national cancer registries in England, Wales, and Scotland, with follow-up completed on February 29, 2020, for England and Wales, and January 31, 2021, for Scotland. Diseases were confirmed using ICD-10 codes, with lung cancer defined as C34. Participants were followed from enrollment until lung cancer diagnosis or censoring, defined as death, study withdrawal, or end of follow-up, whichever occurred first. From 502,354 participants, we sequentially excluded individuals missing baseline plasma proteomic data (N = 449,341), those with prevalent cancer at baseline (N = 3,643), and subjects with incomplete covariate information (N = 15,694), resulting in a final analytical cohort of 33,676 participants. “Prefer not to report” in covariates is also recorded as missing. Additionally, Proteins (GLIPR1, NPM1, PCOLCE) exhibiting > 30% combined missing values or outliers (mean ± 4 standard deviation [sd]) were excluded, while mean imputation was applied to proteins with ≤ 30% missing values (mean missing percent = 10.65%)^[18]^. Propensity score matching (PSM) was performed based on logistic regression-derived propensity scores. Matching covariates included age (years), sex (female, male), ethnic (white people, non-white people), Townsend deprivation index (TDI, continuous variable), and family history of lung cancer (yes, no). TDI was calculated immediately prior to the participant joining the UK Biobank. Based on the preceding national census output areas, each participant was assigned a score corresponding to the output area in which their postcode is located. This index reflects a comprehensive socio-economic level of income, education, housing, employment, and other factors. Multivariable conditional logistic regression models were used to assess associations between circulating proteins and LC risk. Adjustment variables—determined based on scientific rationale and prior publications—included body mass index (BMI), smoking status, alcohol consumption, physical activity, healthy diet, respiratory diseases, and blood glucose. Smoking status is categorized into never, previous, and current smoking. Drinking status was grouped into never, previous, and current drinking. BMI equals weight (kg) / (height squared, m^2^). Regular exercise was defined as more than 75 minutes of vigorous exercise per week or at least one time per week, more than 150 minutes of moderate exercise per week, or a frequency of ≥ 5 times/week^[19]^. Dietary scores were calculated using information on 11 dietary intakes consisting of vegetables (cooked vegetables, raw vegetables), fruits (fresh fruits, dried fruits), oily fish, meat (processed meat, poultry, beef, lamb, pork), and whole grains (**S5** **Table**). A healthy diet is considered to be present when more than four of these dietary intake conditions are met. Respiratory diseases are defined as having more than one of the four diseases: chronic obstructive pulmonary disease, asthma, pulmonary fibrosis, and emphysema. Blood glucose (mmol/L) was one of the biochemical indicators that participants were collected at baseline, measured by hexokinase analysis on a Beckman Coulter AU5800.

Means (± sd) were used to express continuous variables; number of cases (percentage, %) were used to express categorical variables, and paired t-tests or chi-square tests were used for analysis of differences between groups. Association effects were expressed by using OR (95% CI).

**R packages**

MR analysis was conducted with the R package “TwoSampleMR” (version 0.5.6). The mRnd website (https://shiny.cnsgenomics.com/mRnd/) was used to calculate the power of MR analyses. The meta-analysis was conducted with the R package “meta” (version 7.0.0). The SMR and HEIDI tests were performed using SMR software (SMR v1.3.1). R package “coloc” (version 5.2.3) was used in colocalization analyses. CisMR-cML analysis was conducted with the R package “cisMRcML” (version 0.0.0.9000). MR.CUE analysis was conducted with the R package “MR.CUE” (version 1.0). PSM analysis was conducted with the R package “MatchIt” (version 4.7.2). Tests of equilibrium and analysis of differences between groups were performed with the R package “tableone” (version 0.13.2) and “compareGroups” (version 4.8.0). Conditional logistic regression analysis was conducted with the R package “Epi” (version 2.59).

**List of abbreviations**

**MR:** Mendelian randomization; **PRISMA**: Systematic Reviews and Meta-Analysis; **LC**: lung cancer; **OR**: odds ratio; **CI**: confidence interval; **pQTLs**: cis-protein quantitative trait locus; **IVs:** instrumental variables; **cis-****pQTL**: cis-protein quantitative trait loci; **LD**: linkage disequilibrium; **SNP**: single nucleotide polymorphism; **IVW**: inverse variance weighted; **PPH0/1/2/3/4**: the posterior probability of hypothesis 0/1/2/3/4; **MR.CUE**: MR with Correlated horizontal pleiotropy Unraveling shared Etiology and confounding; **HPA**: the Human Protein Atlas; **pTPM**: per million protein-coding genes; **FDR**: false discovery rate; **NPX**: **PPI**: the protein-protein interaction network analysis; Normalized Protein eXpression; **sd**: standard deviation; **PSM**: propensity score matching; **TDI**: Townsend Deprivation Index; **BMI**: body mass index.

**Reference**

1. Page MJ, McKenzie JE, Bossuyt PM, Boutron I, Hoffmann TC, Mulrow CD, et al. The PRISMA 2020 statement: an updated guideline for reporting systematic reviews. BMJ. 2021;372:n71. doi: 10.1136/bmj.n71.

2. Sun J, Zhao J, Jiang F, Wang L, Xiao Q, Han F, et al. Identification of novel protein biomarkers and drug targets for colorectal cancer by integrating human plasma proteome with genome. Genome Med. 2023;15(1):75. doi: 10.1186/s13073-023-01229-9.

3. Papadimitriou N, Dimou N, Tsilidis KK, Banbury B, Martin RM, Lewis SJ, et al. Physical activity and risks of breast and colorectal cancer: a Mendelian randomisation analysis. Nat Commun. 2020;11(1):597. doi: 10.1038/s41467-020-14389-8.

4. Kamat MA, Blackshaw JA, Young R, Surendran P, Burgess S, Danesh J, et al. PhenoScanner V2: an expanded tool for searching human genotype-phenotype associations. Bioinformatics. 2019;35(22):4851-4853. doi: 10.1093/bioinformatics/btz469.

5. Brion MJ, Shakhbazov K, Visscher PM. Calculating statistical power in Mendelian randomization studies. Int J Epidemiol. 2013;42(5):1497-1501. doi: 10.1093/ije/dyt179.

6. Hartwig FP, Davey Smith G, Bowden J. Robust inference in summary data Mendelian randomization via the zero modal pleiotropy assumption. Int J Epidemiol. 2017;46(6):1985-1998. doi: 10.1093/ije/dyx102.

7. Wallace C. Eliciting priors and relaxing the single causal variant assumption in colocalisation analyses. PLoS Genet. 2020;16(4):e1008720. doi: 10.1371/journal.pgen.1008720.

8. Foley CN, Staley JR, Breen PG, Sun BB, Kirk PDW, Burgess S, Howson JMM. A fast and efficient colocalization algorithm for identifying shared genetic risk factors across multiple traits. Nat Commun. 2021;12(1):764. doi: 10.1038/s41467-020-20885-8.

9. Sun J, Luo J, Jiang F, Zhao J, Zhou S, Wang L, et al. Exploring the cross-cancer effect of circulating proteins and discovering potential intervention targets for 13 site-specific cancers. J Natl Cancer Inst. 2023;116(4):565-573. doi: 10.1093/jnci/djad247.

10. Lin Z, Pan W. A robust cis-Mendelian randomization method with application to drug target discovery. Nat Commun. 2024;15(1):6072. doi: 10.1038/s41467-024-50385-y.

11. Yang J, Ferreira T, Morris AP, Medland SE, Madden PA, Heath AC, et al. Conditional and joint multiple-SNP analysis of GWAS summary statistics identifies additional variants influencing complex traits. Nat Genet. 2012;44(4):369-75, s1-3. doi: 10.1038/ng.2213.

12. Cheng Q, Zhang X, Chen LS, Liu J. Mendelian randomization accounting for complex correlated horizontal pleiotropy while elucidating shared genetic etiology. Nat Commun. 2022;13(1):6490. doi: 10.1038/s41467-022-34164-1.

13. Ponten F, Schwenk JM, Asplund A, Edqvist PH. The Human Protein Atlas as a proteomic resource for biomarker discovery. J Intern Med. 2011;270(5):428-446. doi: 10.1111/j.1365-2796.2011.02427.x.

14. Zhou Y, Zhou B, Pache L, Chang M, Khodabakhshi AH, Tanaseichuk O, et al. Metascape provides a biologist-oriented resource for the analysis of systems-level datasets. Nat Commun. 2019;10(1):1523. doi: 10.1038/s41467-019-09234-6.

15. Szklarczyk D, Kirsch R, Koutrouli M, Nastou K, Mehryary F, Hachilif R, et al. The STRING database in 2023: protein-protein association networks and functional enrichment analyses for any sequenced genome of interest. Nucleic Acids Res. 2023;51(D1):D638-D646. doi: 10.1093/nar/gkac1000.

16. Shannon P, Markiel A, Ozier O, Baliga NS, Wang JT, Ramage D, et al. Cytoscape: a software environment for integrated models of biomolecular interaction networks. Genome Res. 2003;13(11):2498-2504. doi: 10.1101/gr.1239303.

17. Sun BB, Chiou J, Traylor M, Benner C, Hsu YH, Richardson TG, et al. Plasma proteomic associations with genetics and health in the UK Biobank. Nature. 2023;622(7982):329-338. doi: 10.1038/s41586-023-06592-6.

18. Xu J, Liu J, Tang J, Liao J, Liu X, Odden MC, Wu C. Plasma proteomic signature of risk and prognosis of frailty in the UK Biobank. Geroscience. 2025;47(2):2365-2381. doi: 10.1007/s11357-024-01415-6.

19. Zhu M, Wang T, Huang Y, Zhao X, Ding Y, Zhu M, et al. Genetic Risk for Overall Cancer and the Benefit of Adherence to a Healthy Lifestyle. Cancer Res. 2021;81(17):4618-4627. doi: 10.1158/0008-5472.Can-21-0836.
