# Supplementary material for: Circulating proteins associated with histological subtypes of lung cancer from genetic and population-based perspectives
Source: PLoS Genet. 2025 Aug 25;21(8):e1011821. doi: 10.1371/journal.pgen.1011821 (PMC12377608; doi:10.1371/journal.pgen.1011821)
Supplement: S1 STROBE MR Checklist — (DOCX) [file pgen.1011821.s003.docx]

**STROBE-MR checklist of recommended items to address in reports of Mendelian randomization studies**^1^ ^2^

| **Item No.** | **Section** | **Checklist item** | **Page No.** | **Relevant text from manuscript** |
| --- | --- | --- | --- | --- |
| 1 | **TITLE and ABSTRACT** | Indicate Mendelian randomization (MR) as the study’s design in the title and/or the abstract if that is a main purpose of the study | Page 2 | Abstract/Line 27-28  *Two-sample Mendelian randomization (MR) analyses* |
|  | **INTRODUCTION** |  |  |  |
| 2 | **Background** | Explain the scientific background and rationale for the reported study. What is the exposure? Is a potential causal relationship between exposure and outcome plausible? Justify why MR is a helpful method to address the study question | Page 4 | Background/Line 77-86  *Proposed biomarkers span a wide range, including single nucleotide polymorphisms (SNPs), circulating tumor DNA methylation, microRNAs, metabolites, and proteins...* |
| 3 | **Objectives** | State specific objectives clearly, including pre-specified causal hypotheses (if any). State that MR is a method that, under specific assumptions, intends to estimate causal effects | Page 5 | Background/Line 103-112  *This study combined data from two extensive proteomic GWAS studies and two large LC GWAS studies to explore the associations between circulating proteins and the risk of LC and its three subtypes.* |
|  | **METHODS** |  |  |  |
| 4 | **Study design and data sources** | Present key elements of the study design early in the article. Consider including a table listing sources of data for all phases of the study. For each data source contributing to the analysis, describe the following: | Page 6-7 | Methods/Data sources Line 136-150  Table 1  *Data sources*  *Summary-level GWAS data for circulating proteins were obtained from two large cohorts: the UK Biobank Pharma Proteomics Project (UKB-PPP) and the deCODE study...* |
|  | a) | Setting: Describe the study design and the underlying population, if possible. Describe the setting, locations, and relevant dates, including periods of recruitment, exposure, follow-up, and data collection, when available. | Page 6-7 | Methods/Data sources Line 136-150  *the UK Biobank Pharma Proteomics Project (UKB-PPP) and the deCODE study. The UKB-PPP study examined plasma proteins in 54,219 participants with an average age of 57 years and publicly available GWAS data for 2,940 proteins...* |
|  | b) | Participants: Give the eligibility criteria, and the sources and methods of selection of participants. Report the sample size, and whether any power or sample size calculations were carried out prior to the main analysis | S1 Text, Page 2 | Line 40-44  *Power calculations of MR analyses*  *We used the mRnd online tool (https://shiny.cnsgenomics.com/mRnd/) to calculate the statistical power of MR analyses[4].* |
|  | c) | Describe measurement, quality control and selection of genetic variants | S1 Text, Page 2 | Line 24-39  Instrumental variables (IVs) selection  *We strictly followed the three basic assumptions of MR analysis to screen for the IVs for proteins...* |
|  | d) | For each exposure, outcome, and other relevant variables, describe methods of assessment and diagnostic criteria for diseases | - | Not available. |
|  | e) | Provide details of ethics committee approval and participant informed consent, if relevant | Page 6 | Line 115-123  *Ethics Statement*  *The original studies of GWAS data used in this study have been approved by relevant ethical review institutions and informed consent of participants...* |
| 5 | **Assumptions** | Explicitly state the three core IV assumptions for the main analysis (relevance, independence and exclusion restriction) as well assumptions for any additional or sensitivity analysis | S1 Text, Page 2 | Line 24-39  *Instrumental variables (IVs) selection*  *We strictly followed the three basic assumptions of MR analysis to screen for the IVs for proteins...* |
| 6 | **Statistical methods: main analysis** | Describe statistical methods and statistics used |  |  |
|  | a) | Describe how quantitative variables were handled in the analyses (i.e., scale, units, model) | Not available. | Not available. |
|  | b) | Describe how genetic variants were handled in the analyses and, if applicable, how their weights were selected | S1 Text, Page 2 | Line 24-39  *Instrumental variables (IVs) selection*  *We strictly followed the three basic assumptions of MR analysis to screen for the IVs for proteins...* |
|  | c) | Describe the MR estimator (e.g. two-stage least squares, Wald ratio) and related statistics. Detail the included covariates and, in case of two-sample MR, whether the same covariate set was used for adjustment in the two samples | Page 2 and S7-S22 Table | Abstract/ Line 36-44; S7-22 Table  *The results showed that twenty-five proteins were significantly associated with LC or its subtypes, including 15 novel findings.* |
|  | d) | Explain how missing data were addressed | Not available. | Not available. |
|  | e) | If applicable, indicate how multiple testing was addressed | Page 9 | Methods/Line 169-170  *The Benjamini-Hochberg method was used to adjust for multiple tests.* |
| 7 | **Assessment of assumptions** | Describe any methods or prior knowledge used to assess the assumptions or justify their validity | Page 9 | Methods/Line 161-165  *When only one genetic variant was available, analysis was performed using the Wald ratio method. When two or more genetic variants were present, the analysis was performed using the inverse variance weighted (IVW) method of the random-effects model.* |
| 8 | **Sensitivity analyses and additional analyses** | Describe any sensitivity analyses or additional analyses performed (e.g. comparison of effect estimates from different approaches, independent replication, bias analytic techniques, validation of instruments, simulations) | Page 9 and Page 10-11 | Methods/Line 164-165 and 174-212  *Meanwhile, MR-Egger, weighted median, weighted mode, and simple mode were used for supplementary analyses*  *MR_meta, reverse_MR, SMR, colocalization, cisMR-cML, MR.CUE and phenotype scanning analyses to identify significant proteins.* |
| 9 | **Software and pre-registration** |  |  |  |
|  | a) | Name statistical software and package(s), including version and settings used | S1 Text, Page 8 | Line 167-178  *R packages*  *MR analysis was conducted with the R package “TwoSampleMR” (version 0.5.6).* |
|  | b) | State whether the study protocol and details were pre-registered (as well as when and where) | Not available. | Not available. |
|  | **RESULTS** |  |  |  |
| 10 | **Descriptive data** |  |  |  |
|  | a) | Report the numbers of individuals at each stage of included studies and reasons for exclusion. Consider use of a flow diagram | Fig 1 | Fig 1 |
|  | b) | Report summary statistics for phenotypic exposure(s), outcome(s), and other relevant variables (e.g. means, SDs, proportions) | S3, S4, and S6 Tables | S3, S4, and S6 Tables |
|  | c) | If the data sources include meta-analyses of previous studies, provide the assessments of heterogeneity across these studies | Not available. | Not available. |
|  | d) | For two-sample MR:  i. Provide justification of the similarity of the genetic variant-exposure associations between the exposure and outcome samples  ii. Provide information on the number of individuals who overlap between the exposure and outcome studies | Page 13 | Results/Line 253-258  *After screening and harmonizing with outcome data, the two studies covered a total of 879 overlapping proteins, with an additional 1,062 unique proteins provided by UKB-PPP and 792 unique proteins supplied by the deCODE study, resulting in a total of 2,733 unique predicted proteins analyzed. The F-statistics of the instrumental variables for these proteins were all higher than 10. The proportion of variance in exposure explained by a single genetic variant ranged from 0.0004 to 0.387, as outlined in S6 Table.* |
| 11 | **Main results** |  |  |  |
|  | a) | Report the associations between genetic variant and exposure, and between genetic variant and outcome, preferably on an interpretable scale | Page 13 | Results/Line 253-258  *After screening and harmonizing with outcome data, the two studies covered a total of 879 overlapping proteins, with an additional 1,062 unique proteins provided by UKB-PPP and 792 unique proteins supplied by the deCODE study, resulting in a total of 2,733 unique predicted proteins analyzed. The F-statistics of the instrumental variables for these proteins were all higher than 10. The proportion of variance in exposure explained by a single genetic variant ranged from 0.0004 to 0.387, as outlined in S6 Table.* |
|  | b) | Report MR estimates of the relationship between exposure and outcome, and the measures of uncertainty from the MR analysis, on an interpretable scale, such as odds ratio or relative risk per SD difference | Page 2 and S7-23 Table | Line 40-44, S7-23 Table  *RPL14 exhibited positive associations with overall LC (MR_meta: odds ratio [OR]: 2.012, 95% confidence interval [CI]: 1.297–3.119; UKB: OR: 1.509, 95% CI: 1.015–2.244). Similarly, AGER showed significant protective effects against LUSC risk (MR_meta: OR: 0.572, 95%CI: 0.368–0.889; OR: 0.366, 95% CI: 0.158–0.850).* |
|  | c) | If relevant, consider translating estimates of relative risk into absolute risk for a meaningful time period | Not available. | Not available. |
|  | d) | Consider plots to visualize results (e.g. forest plot, scatterplot of associations between genetic variants and outcome versus between genetic variants and exposure) | Fig 2-4 | Fig 2-4 |
| 12 | **Assessment of assumptions** |  |  |  |
|  | a) | Report the assessment of the validity of the assumptions | Page 13 | Results/Line 259-267  *A total of 59 unique plasma proteins fulfilling the criteria for candidate protein screening were identified. Among these, 27 genetically predicted proteins were causally associated with LC risk, 14 with LUSC, 14 with LUAD, and eight with SCLC (Fig 2, S23 Table). .* |
|  | b) | Report any additional statistics (e.g., assessments of heterogeneity across genetic variants, such as *I^2^*, Q statistic or E-value) | Page 13 and S24 Table | Results/Line 259-267and S24 Table  *No evidence of heterogeneity or pleiotropy was observed (p > 0.05). Steiger directionality tests confirmed that the direction of causality consistently pointed from proteins to LC risk. Most of these analyses achieved a statistical power of up to 80%, as detailed in S24 Table.* |
| 13 | **Sensitivity analyses and additional analyses** |  |  |  |
|  | a) | Report any sensitivity analyses to assess the robustness of the main results to violations of the assumptions | Page 13 and S24 Table | Results/Line 259-267 and S24 Table  *No evidence of heterogeneity or pleiotropy was observed (p > 0.05). Steiger directionality tests confirmed that the direction of causality consistently pointed from proteins to LC risk. Most of these analyses achieved a statistical power of up to 80%, as detailed in S24 Table.* |
|  | b) | Report results from other sensitivity analyses or additional analyses | Page 13-15 | Results/Line 259-320  *No evidence of heterogeneity or pleiotropy was observed (p > 0.05). Steiger directionality tests confirmed that the direction of causality consistently pointed from proteins to LC risk.* |
|  | c) | Report any assessment of direction of causal relationship (e.g., bidirectional MR) | Page 14 | Results/Line 281-286  *The number of IVs for overall LC, LUSC, LUAD, and SCLC from the FinnGen database was 7, 2, 1, and 4, respectively, while the number of IVs from the TRICL database was 14, 7, 10, and 2. All IVs exhibited F-statistics greater than 10, with single-SNP R² values ranging from 0.004 to 0.046. Reverse MR analyses with LC as the exposure and protein levels as the outcome, suggested that protein PLAU may be affected by LUAD (S26 Table). To avoid the potential reverse causality effect, this protein was excluded from subsequent analyses.* |
|  | d) | When relevant, report and compare with estimates from non-MR analyses | Page 15-17 | Results/Line 300-362  *Notably, the colocalization analysis provided strong support for two associations: AGER with LUSC (PPH4 = 0.821) and CTBS with LUAD (PPH4 = 0.785), indicating a high probability of shared causal variability between protein levels and LC risk* |
|  | e) | Consider additional plots to visualize results (e.g., leave-one-out analyses) |  | *No, but heterogeneity tests were performed.* |
|  | **DISCUSSION** |  |  |  |
| 14 | **Key results** | Summarize key results with reference to study objectives | Page 17-18 | Discussion/Line 364-375  *This study conducted comprehensive MR analyses to explore causal associations between a total of 2,733 unique genetically predictive proteins from the UKB-PPP and deCODE study with LC and its three subtypes.* |
| 15 | **Limitations** | Discuss limitations of the study, taking into account the validity of the IV assumptions, other sources of potential bias, and imprecision. Discuss both direction and magnitude of any potential bias and any efforts to address them | Page 22-23 | Discussion/Line 493-516  *This study has several limitations. First…* |
| 16 | **Interpretation** |  |  |  |
|  | a) | Meaning: Give a cautious overall interpretation of results in the context of their limitations and in comparison with other studies | Page 22-23 | Discussion/Line 493-516  *This study has several limitations. … Finally, while this evidence strengthens our understanding of potential causal associations, these findings should be interpreted cautiously and further validated through experimental studies and large-scale population-based investigations.* |
|  | b) | Mechanism: Discuss underlying biological mechanisms that could drive a potential causal relationship between the investigated exposure and the outcome, and whether the gene-environment equivalence assumption is reasonable. Use causal language carefully, clarifying that IV estimates may provide causal effects only under certain assumptions | Page 23 | Discussion/Line 514-516  *Finally, while this evidence strengthens our understanding of potential causal associations, these findings should be interpreted cautiously and further validated through experimental studies and large-scale population-based investigations.* |
|  | c) | Clinical relevance: Discuss whether the results have clinical or public policy relevance, and to what extent they inform effect sizes of possible interventions | Not available. | Not available. |
| 17 | **Generalizability** | Discuss the generalizability of the study results (a) to other populations, (b) across other exposure periods/timings, and (c) across other levels of exposure | Page 22 | Discussion/Line 493-494  *First, European-focused GWAS data limited sex/age-stratified analyses and cross-ethnic generalizability.* |
|  | **OTHER INFORMATION** |  |  |  |
| 18 | **Funding** | Describe sources of funding and the role of funders in the present study and, if applicable, sources of funding for the databases and original study or studies on which the present study is based | Page 26 | Declarations/Funding Line 577-583  *This work was supported by National Natural Science Foundation of China (82430107 to KXC; 82204121 to ZYL)...* |
| 19 | **Data and data sharing** | Provide the data used to perform all analyses or report where and how the data can be accessed, and reference these sources in the article. Provide the statistical code needed to reproduce the results in the article, or report whether the code is publicly accessible and if so, where | Page 25-26 | Declarations/Line 540-573  *Availability of data and materials* |
| 20 | **Conflicts of Interest** | All authors should declare all potential conflicts of interest | Page 26 | Declarations/Line 581-582  *Competing interests*  *The authors have declared that no competing interests exist.* |

This checklist is copyrighted by the Equator Network under the Creative Commons Attribution 3.0 Unported (CC BY 3.0) license.

1. Skrivankova VW, Richmond RC, Woolf BAR, Yarmolinsky J, Davies NM, Swanson SA, et al. Strengthening the Reporting of Observational Studies in Epidemiology using Mendelian Randomization (STROBE-MR) Statement. JAMA. 2021;under review.

2. Skrivankova VW, Richmond RC, Woolf BAR, Davies NM, Swanson SA, VanderWeele TJ, et al. Strengthening the Reporting of Observational Studies in Epidemiology using Mendelian Randomisation (STROBE-MR): Explanation and Elaboration. BMJ. 2021;375:n2233.
