# Supplementary material for: Circulating proteins associated with histological subtypes of lung cancer from genetic and population-based perspectives
Source: PLoS Genet. 2025 Aug 25;21(8):e1011821. doi: 10.1371/journal.pgen.1011821 (PMC12377608; doi:10.1371/journal.pgen.1011821)
Supplement: S1 Fig — (LC: lung cancer; LUSC: lung squamous carcinoma; LUAD: lung adenocarcinoma; SCLC: small cell lung cancer; BMI: body mass index; TDI: Thompson Deprivation Index). (S1_Fig.DOCX) [file pgen.1011821.s004.docx]

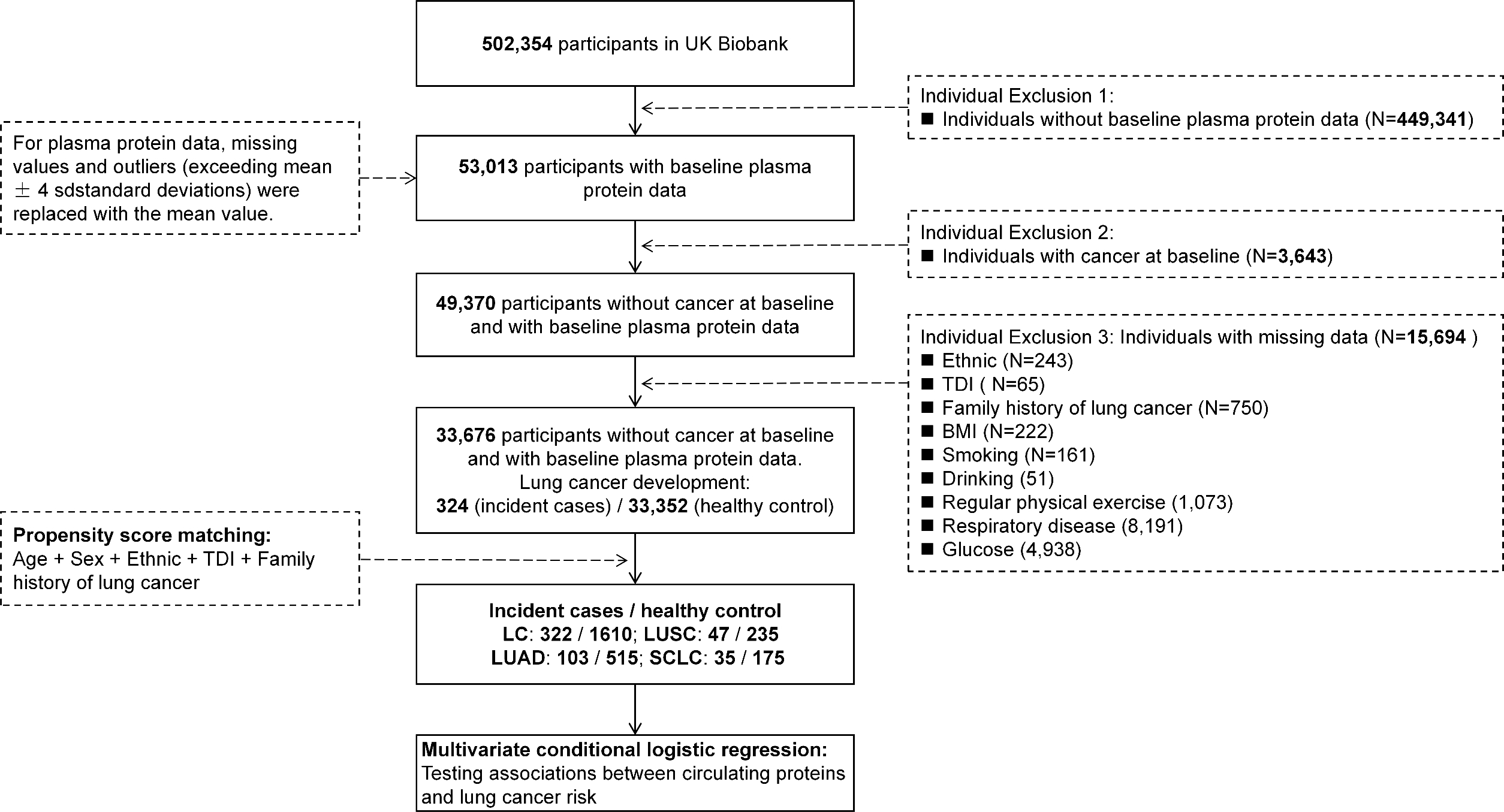
Fig S1. Flowchart of study population selection in UK Biobank

(LC: lung cancer; LUSC: lung squamous carcinoma; LUAD: lung adenocarcinoma; SCLC: small cell lung cancer; BMI: body mass index; TDI: Thompson Deprivation Index)
